# Deamination of 5-Methylcytosine Residues in Mammalian Cells

**Published:** 2009-10

**Authors:** E.V. Gromenko, P.V. Spirin, E.A. Kubareva, E.A. Romanova, V.S. Prassolov, O.V. Shpanchenko, O.A. Dontsova

**Affiliations:** 1 Department of Chemistry, Lomonosov Moscow State University;; 2 Engelhardt Institute of Molecular Biology, Russian Academy of Sciences;; 3 Belozersky Institute of Physico-Chemical Biology, Lomonosov Moscow State University

## Abstract

DNA demethylation in mammalia occurs after fertilization and during embryogenesis and accompanies cell aging and cancer transformation. With the help of the primer extension reaction, MALDI MS and DNA cleavage by thymine DNA glycosylase deamination of 5-methylcytosine residues has been shown to take place when the model methylated DNA duplexes are treated with nuclear extracts from the cell lines CHO, HeLa, and Skov3. The hypothesis that deamination of 5-methylcytosine is the first stage of demethylation in mammalia has been postulated.

## INTRODUCTION

Active DNA demethylation in mammalia is necessary for the proper development of the organism, formation of the immune response, and memory. DNA demethylation accompanies the emergence of various diseases and aging.

Global demethylation is observed in the paternal pronuclei in the embryos of mice [1], rats, pigs, cows, and humans [2], as well as in their germ cells. It provides epigenetic reprogramming and specific gene expression.

Methylation and demethylation of DNA in the cells of the nervous system has an influence on synaptic plasticity and memory formation. DNA methylation is necessary for the inactivation of the gene pp1 that suppresses memory processes; whereas active DNA demethylation is associated with activation of the gene reelin that promotes the formation of memory in rats [3]. Also, active DNA demethylation is necessary for normal neurogenesis in the embryos of fish Danio rerio. Suppression of the expression of protein Gadd45a (DNA-damage-inducible protein 45 alpha) or other proteins involved in the process of demethylation leads to the death of neurons as a result of hypermethylation and suppression of the genes responsible for neurogenesis [4].

Methylation and demethylation of DNA is an important part of epigenetic control during the immune response [5]. Demethylation of the promoter regions of the genes of cytokines il-2 and ifn-≤ as a result of CD8 T cells contact with antigens leads to rapid expression of cytokines [6-8].

DNA demethylation accompanies cell aging [9]. A difference in the degree of demethylation in different tissues has been shown in rats. The degree of demethylation is higher in brain tissues than in liver tissues. Age-related decrease in the content of 5-methylcytosine in DNA was also found in such cells as lung and skin fibroblasts: for the latter case, the connection of demethylation to reduced growth potential in culture was shown [10].

Global demethylation of the genome is observed in all examined cancer cells [11-13]. Hypomethylated sites turned out to be numerous repeats, imprinted genes, tissue-specific genes, oncogenes, and genes associated with the processes of invasion and metastasis of tumors [14, 13]. At the same time, some loci, including many tumor-suppressor genes, are hypermethylated, which results in suppression of their expression [12, 15, 16].

It is clear that DNA demethylation plays an important role in the life of cells, while the mechanism of DNA demethylation and the participants in this process in mammalia have yet to be identified. The aim of this work was to study the mechanism of DNA demethylation in mammalian cells.

## MATERIALS | METHODS

Synthesis of oligodeoxyribonucleotides. Oligonucleotides I-III were synthesized using the amido-phosphite method in automatic mode on a synthesizer manufactured by Applied Biosystems, with commercial reagents and solvents according to the standard protocol:

5’-CATGTCTAACmCGmCGmCGAGAAATGGTAATGTATGGAGT* (I)

5’-CATACATTACCATTTCTmCGmCGmCGGTTAGACATGGC* (II)

5’-CATACATTACCATTTC (III), where * is the amino group bound to the 3’-end of oligodeoxyribonucleotides by the linker.

Oligonucleotides

5’-TT(Biotin-T)TTTTTTTGTCTACGATCGAACmCGmCGmCGAGAAGCTTGTAT* (IV), 5’-ATACAAGCTTCTmCGmCGmCGGTTCGATCGTAGACAAAAAAAAAA* (V) - commercial preparations ("Synthol").

Cultivation of cell lines and preparation of nuclear extracts. Continuous cell lines of Chinese hamster ovary (CHO), a human cervical carcinoma (HeLa), and human ovarian adenocarcinoma (Skov3) were grown in a standard DMEM medium containing 10% fetal serum (FCS), 4 mM L-glutamine, 1 mM sodium pyruvate, a streptomycin/penicillin concentration of 100 ≤g/ml and 100 U/ml, respectively, at 37° C in an atmosphere of 5% CO_2_. For subculturing, the cell monolayer was washed with a PBS buffer (10 mM Na_2_HPO_4_, 1.5 mM KH_2_PO_4_, 137 mM NaCl, 2.7 mM KCl, pH 7.4), a standard solution of trypsin-EDTA (Sigma) was added, and culture bottles were placed in a CO_2_ incubator for 3-5 minutes. Then, a medium with FCS was added, and cells were suspended by pipetting. The cells were then placed in the necessary number of culture bottles. The cells grown to the monolayer were collected by centrifugation at 2300 rpm for 10 minutes at 4° C. They were then washed several times with a PBS buffer and lysed in buffer L (20 mM Hepes (pH 7.6), 10 mM NaCl, 1.5 mM MgCl_2_, 20% glycerol, 0.1% Triton X100, 1 mM DTT, cocktail of protease inhibitors). After 2 minutes, the lysate was suspended and centrifuged for 20-30s at 10,000 rpm. An equal volume of buffer NE (20 mM Hepes (pH 7.6), 500 mM NaCl, 1.5 mM MgCl_2_, 20% glycerol, 0.1% Triton X100, 1 mM DTT, a cocktail of protease inhibitors) was added to the pellet, then it was carefully suspended and placed on a shaker for 30-60 minutes. Then, lysate was centrifuged for 10 minutes at 10 000 rpm.

Formation of DNA-duplex and treatment with nuclear extracts from cells. Formation of DNA-duplex I/II proceeded on a PCR thermal cycler by keeping 20 ≤l of the reaction mixture containing 500 pmol of oligonucleotide I and 550 pmol of oligonucleotide II at 95° C for 3 minutes and then cooling the thermostat from 95 to 35° C at a speed of 0.5°/min and from 35 to 20° C at a speed of 0.25°/min.

Then, 50 ≤l of the reaction mixture containing buffer NE, 10 mM of DNA-duplex I/II, and the nuclear extract corresponding to ~ 50000 cells was incubated at 37° C for 1 hour. After a three-fold phenol deproteinization, the DNA duplex was precipitated with 2.5 volumes of ethanol, with the addition of a 1/10 volume of 3 M NaOAc (pH 5.5) by keeping for 2 hours at -20° C. The pellet was precipitated by centrifugation at 14000 rpm for 10 minutes, washed with 70% ethanol, dried in vacuum, and dissolved in 20 ≤l of water.

Primer extension reaction. A radioactive label was introduced at the 5’-end of primer III in advance. To do this, a 20 ≤l reaction mixture containing a buffer solution for T4 polynucleotide kinase (500 mM Tris-HCl (pH 7.6), 100 mM MgCl2, 50 mM DTT, 1 mM spermidine, 1 mM EDTA, 1 mM ADP), 10 mM of primer III, 5 units of T4 polynucleotide kinase (MBI Fermentas, 10 units/≤l) and 10 mM [≤-^32^P] ATP (GE Healthcare, the specific activity of 220 TBq/mmol or 6000 Ci/mmol) was incubated at 37° C for 1 hour. The enzyme was heat-inactivated for 15 minutes at 75° C. The ^32^P-labeled primer III was purified by electrophoresis in denaturing 12% PAAG. The area containing primer III with the radioactive label was visualised using autoradiography and excised. Primer III was eluted from the gel by adding 400 ≤l of a GES buffer (10 mM Tris-HCl (pH 8.0), 1% SDS, 0.5% Triton X100, 50 mM EDTA) supplemented with 400 ≤l of phenol with vigorous stirring overnight. The aqueous phase was separated by centrifugation at 14000 rpm for 5 minutes, primer III was precipitated as described above and was dissolved in 20 ≤l of water. The amount of primer III was evaluated either by radioactivity or spectrophotometrically by the absorption of the solution at 260 nm. After that, 4 ≤l of the solution of DNA-duplex I/II (2.5 mM) and 1 ≤ of a 10X reaction buffer (500 mM Tris-HCl (pH 8.0), 50 mM MgCl_2_, 10 mM DTT) were added to 3 ≤l of a purified ^32^P-labeled primer III (3.3 mM). Hybridization was performed on the PCR thermal cycler by cooling the thermostat from 70 to 42° C at a speed of 0.4°/min with keeping a constant temperature for 21 seconds and increasing the time of every subsequent cycle by 1 second. Then, 5 units of Klenow fragment without exonuclease activity (MBI Fermentas, 10 units/ul) and 1 ≤l of a mixture of dATP, dCTP, dTTP (1 mM each), and ddNTP (0.01 mM) were added to the reaction mixture. The mixture was incubated at 37° C for 10 minutes. The reaction was stopped by heating to 75° C for 10 minutes. Analysis of the products of the primer extension reaction was carried out by electrophoresis in denaturing 10% PAAG. The gel was dried in vacuum, exposed to the screen of a BAS CASSETTE 2340, and information from the screen was read on the FUJIFILM FLA 3000 using the BASReader 3.14 computer programm.

Treatment of DNA duplexes by thymine DNA glycosylase (TDG). A 20 ≤l reaction mixture containing 0.5 mM of DNA duplex I/II, ^32^P-labeled at one of the DNA strands, 10 units of TDG (R Amp; D Systems, 5 U/≤l), and the buffer (10 mM Hepes (pH 7.4), 100 mM KCl, 10 mM EDTA) was incubated at 65° C for 1 hour. Then, a 10 ≤l of 3-fold alkaline buffer (300 mM NaOH, 97% formamide, 0.2% bromphenol blue) was added to the reaction. It was incubated at 95° C for 10 minutes and then rapidly cooled to 2-8° C. The reaction products were analyzed by electrophoresis in 20% PAAG. The gel was dried using a vacuum dryer and exposed to the screen of a BAS CASSETTE 2340. Information from the screen was read on the FUJIFILM FLA 3000 using the BASReader 3.14 program.

Analysis of DNA fragments using mass spectrometry. The biotinylated DNA duplex IV/V was formed as described above for the duplex I/II. Then, it was treated with nuclear extracts from cells, and purification on streptavidin-Sepharose was performed. Streptavidin-Sepharose was pre-equilibrated in buffer R (100 mM Tris-HCl (pH 7.5), 10 mM MgCl_2_, 100 mM KCl, 0.1 mg/ml BSA). For that, 100 ≤l of a 50% suspension of streptavidin-Sepharose was mixed with 200 ≤l of buffer R and incubated with weak stirring for 10-15 minutes. Streptavidin-Sepharose was precipitated by centrifugation at 3000 rpm for 3-4 minutes, the buffer was replaced, and beads were resuspended. The procedure described above was repeated 5 times. A 500 pmol of biotinylated DNA duplex IV/V was added to the equilibrated Sepharose. The binding of the duplex with Sepharose was carried out under mild stirring at 4° C for 2-12 hours. Then, the resin was washed 6 times by 1 ml of buffer R, as described above. After that, 30 units of restriction endonucleases Hind≤≤≤ (MBI Fermentas, 10 U/≤l) and Pvu≤ (MBI Fermentas, 10 U/≤l) were added to the reaction mixture and it was kept with low stirring at 37° C for 16 hours. Streptavidin-Sepharose was sedimented by centrifugation at 5000 rpm for 5 minutes. The obtained fragments were separated by electrophoresis in denaturing 20% PAAG. The gel was stained with SYBR Green in a buffer solution TBE (100 mM Tris-HCl, 100 mM H_3_BO_3_, 2 mM EDTA). The analyzed fragment was eluted from the gel using the method described for the primer. Then, samples were concentrated and desalted on ZipTip C18 (Millipore). The resin was washed with a 50 mM aqueous solution of ammonium citrate, and the oligonucleotide was eluted with a solution of 25 mM ammonium citrate in 50% acetonitrile. On the matrix for MALDI MS 1 ≤l of the sample, 0.5 ≤l of a 50 mM solution of ammonium citrate and 0.5 ≤l of 3-hydroxypicoline acid (Fluka, 20 mg / ml in acetonitrile) were mixed, then the mixture was dried in air. Mass spectra were obtained on the MALDI TOF/TOF mass spectrometer Ultraflex II BRUKER (Germany), equipped with a UV laser (Nd). Mass spectra were obtained in the mode of positive ions in linear fashion.

## DISCUSSION OF RESULTS | CONCLUSIONS

To study the process of DNA demethylation, complementary oligodeoxynucleotides I and II, each containing 3 residues of 5-methylcytosine in the context of dinucleotide 5’-mCpG, which is typical for the mammalian genome, were designed and synthesized. For the protection against the nucleases present in the cell lysates 3’-protruding ends of DNA-duplex I/II carried protective groups.

**Fig. 1.**
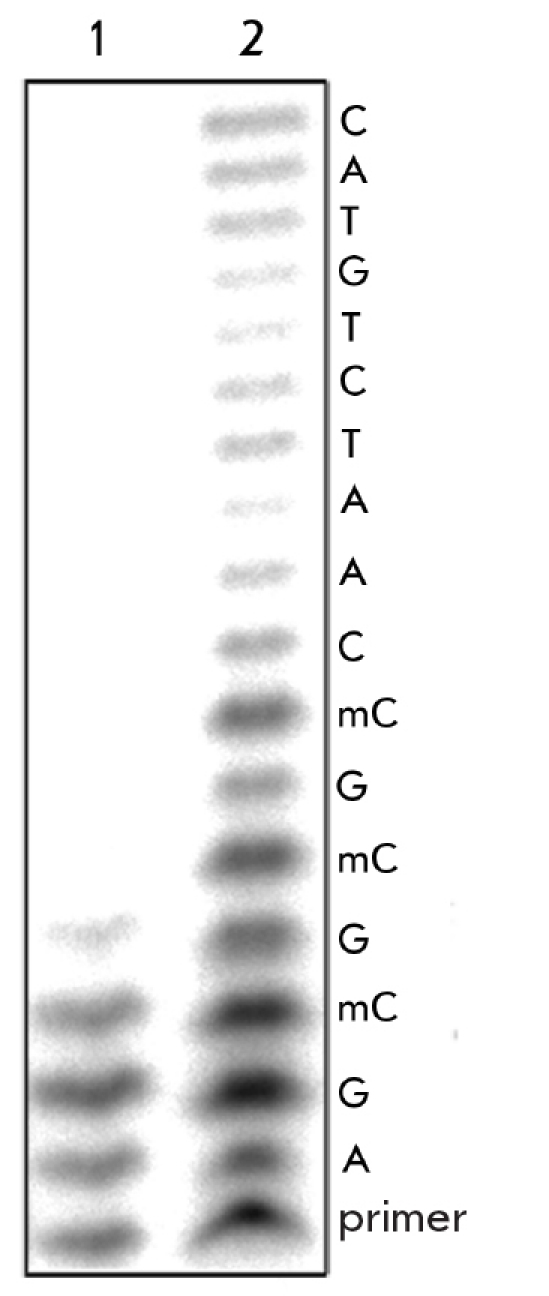
Primer extension reaction without dGTP for methylated oligodeoxyribonucleotide duplex treated with buffer for extracts (control) (1), or nuclear extract from CHO cell line (2). The partial sequence of model oligodeoxyribonucleotide I is shown on the right

The extension of primer III was used to monitor changes in the nucleotide sequence. Primer III is complementary to the 3’-terminal region of oligonucleotide I. In the absence of dGTP in the medium and in the presence of ddNTP, the primer had to be extended up to the first 5-methylcytosine residue (lane 1, Fig. 1). After the treatment of DNA-duplex I/II with nuclear extracts from the CHO cell line longer primer-extension products appear (lane 2, Fig. 1). This phenomenon could be explained by the conversion of 5-methylcytosine into thymine; i.e., the deamination process takes place when DNA-duplex I/II is treated with the extract of CHO cells.

**Fig. 2.**
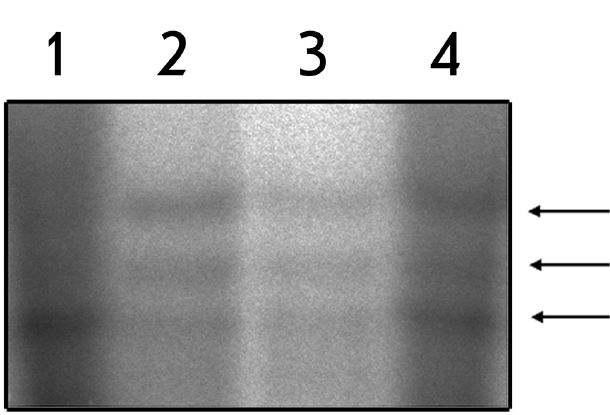
The action of thymine DNA glycosylase on methylated duplex I/II treated with buffer for extracts (control) (1), or nuclear extracts from cell lines CHO (2), HeLa (3), and Skov3 (4). The positions of generated DNA fragments are indicated by arrows

To test this hypothesis, DNA-duplex I/II with three 5’-mCpG sites was sequentially treated with the nuclear extract of cell lines (CHO, HeLa or Skov3) and thymine DNA glycosylase, which recognizes T/G-mismatch and cuts the DNA strand containing T (Fig. 2, lanes 2, 3, 4, respectively). After the separation of reaction products, new bands corresponding to the position of 5-methylcytosine residues could be detected by autoradiography. That indicates the deamination of 5-methylcytosine residues.

Deamination of DNA duplexes after the treatment with the nuclear extracts of the cells was shown by MALDI MS. For that, we used oligodeoxynucleotide duplex IV/V containing the dinucleotides 5’-mCpG, endonuclease Hind≤≤≤ and Pvu≤ recognition sites, flanking the analyzed fragment (underlined), as well as biotin at the 5’-end for the affinity isolation of the DNA duplex on streptavidin-Sepharose.

**Fig. 3.**
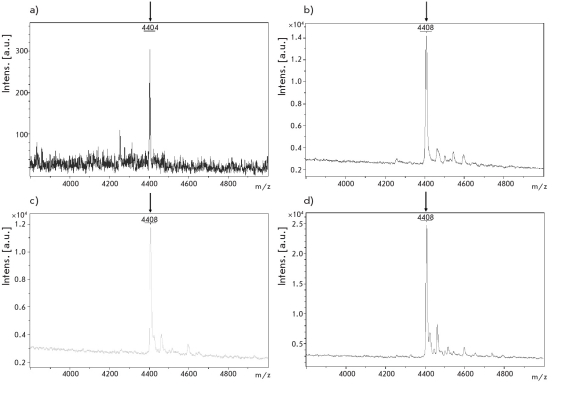
MALDI massspectra obtained for methylated DNA fragment treated with buffer for extracts (control) (a), or nuclear extracts from cell lines CHO (b), HeLa (c), Skov3 (d)

The size of the fragment containing three 5’-mCpG sites is 14 nucleotides; its calculated molecular mass is 4404.8 Da (Fig. 3, a).

The spectra of the analyzed fragment derived from the DNA duplex after the treatment with nuclear extracts from cell lines (CHO, HeLa, or Skov3) have a signal at 4408 Da (Fig. 3, b-d), corresponding to deamination of three 5-methylcytosine residues.

Cell lines CHO, HeLa, and Skov3 are derived from cancer cells in which global demethylation of the genome is known to occur. One can suggest that, the deamination of 5-methylcytosine observed as a result of the treatment with nuclear extracts of these cell lines is the first stage of the DNA demethylation in mammalia. It is known that in Danio rerio embryos DNA demethylation is a multistep process involving deamination of 5-methylcytosine with the deaminases AID (Activation Induced deaminase)/Apobec (Apolipo-protein B RNA-editing catalytic component), removal of thymine by glycosylase MBD4, and BER-repair [4, 17]. In mammalian cells, there are suitable candidates from the cytosine deaminases family - AID and Apobec1, which are co-expressed with pluripotent genes in oocytes, embryonic germ cells, and embryonic stem cells [18]. However, it is known that these enzymes perform deamination only in single-stranded RNA (Apobec1) or DNA (AID). In addition, AID and Apobec deaminate cytosine residues much more effectively than 5-methylcytosine. DNA methyltransferase Dnmt3b can also perform the role of 5-methyl cytosine deaminase when the concentration of S-adenosylmethionine (AdoMet) is low [19], but this reaction takes place with very low efficiency, making its participation in the global demethylation unlikely. Moreover, the concentration of AdoMet in the cell is usually quite high.

A good candidate for the role of thymine glycosylase is MBD4 glycosylase, which contains methyl-binding and glycosylase domains and removs of thymine from T/G-mismatch [20]. Although MBD4 deficient mice are viable, mutations in the CpG-sites occur more frequently in such mice [21]. MBD4 removes thymine from T/G-mismatch with the formation of apurine site that is immediately cut by AP endonuclease. In the symmetrically methylated CpG-sites, the deamination of both DNA strands should lead to the formation of the TG/GT region. If MBD4 recognizes such a region then, after the action of AP endonuclease, a double break should form, which would lead to the loss of genetic material. In addition, MBD4 removes uracil from U/G-mismatch faster than thymine from T/G [20].

Another mechanism of DNA demethylation is also known. It is observed in plant Arabidopsis thaliana and involves the direct removal of the 5-methylcytosine base using bifunctional glycosylases/liases (ROS1, DML2, DML3, DME) followed by BER-repair [22-25]. There are two suitable glycosylase in mammalia: thymine DNA glycosylase (TDG) and methyl-binding protein MBD4. However, both TDG and MBD4 have weak 5-methylcytosine glycosylase activity compared to the ability to excise thymine [26]. In addition, active demethylation of paternal chromatin was observed in mouse embryos with a knocked-out mbd4 gene [21].

It is worth mentioning other theoretically possible mechanisms of 5-methylcytosine demethylation. This is the direct removal of methyl groups with the formation of cytosine and excision of one or more nucleotides containing 5-methylcytosine (NER-repair). In vertebrates, there are orthologs of bacterial demethylase (oxidoreductase) AlkB responsible for direct removal of methyl groups from 1-methyladenine and 3-methylcytosine in prokaryotes [2]. However, there is no data indicating that any of the orthologs can carry demethylation of 5-methylcytosine. Histone demethylases (HDMs) are homologous to bacterial AlkB [27], but none of them is involved in DNA demethylation. Earlier, it was shown that methyl-binding protein MBD2b can directly remove the methyl group of 5-methylcytosine with the formation of C and methanol as the reaction products [28]. However, this result could not be reproduced in other laboratories. Moreover, mice lacking MBD2b had a normal phenotype and normal pattern of DNA methylation. As for the mechanism of DNA demethylation by NER-repair, there is no evidence of the occurrence of the process in vivo.

Thus, further study of DNA demethylation in mammals is necessary to understand the mechanism of this essential life process. 

## Acknowledgements

The work was supported by the Russian Foundation for Basic Research (grant № 07-04-00545) in part and the Charity Fund "Science for the Extension of Life". The authors are grateful to Prof. M.V. Serebryakova for the MALDI MS analysis.
